# Evaluation of the Prognostic Relevance of Differential Claudin Gene Expression Highlights Claudin-4 as Being Suppressed by TGFβ1 Inhibitor in Colorectal Cancer

**DOI:** 10.3389/fgene.2022.783016

**Published:** 2022-02-24

**Authors:** Linqi Yang, Wenqi Zhang, Meng Li, Jinxi Dam, Kai Huang, Yihan Wang, Zhicong Qiu, Tao Sun, Pingping Chen, Zhenduo Zhang, Wei Zhang

**Affiliations:** ^1^ Department of Pharmacology, Hebei University of Chinese Medicine, Shijiazhuang, China; ^2^ Department of Hematology, The Fourth Hospital of Hebei Medical University, Shijiazhuang, China; ^3^ College of Natural Science, Michigan State University, East Lansing, MI, United States; ^4^ Shijiazhuang People’s Hospital, Shijiazhuang, China

**Keywords:** colorectal cancer, CLDNs, public databases, TGFβ1, molecular docking, prognostic value

## Abstract

**Background:** Claudins (CLDNs) are a family of closely related transmembrane proteins that have been linked to oncogenic transformation and metastasis across a range of cancers, suggesting that they may be valuable diagnostic and/or prognostic biomarkers that can be used to evaluate patient outcomes. However, CLDN expression patterns associated with colorectal cancer (CRC) remain to be defined.

**Methods:** The mRNA levels of 21 different CLDN family genes were assessed across 20 tumor types using the Oncomine database. Correlations between these genes and patient clinical outcomes, immune cell infiltration, clinicopathological staging, lymph node metastasis, and mutational status were analyzed using the GEPIA, UALCAN, Human Protein Atlas, Tumor Immune Estimation Resource, STRING, Genenetwork, cBioportal, and DAVID databases in an effort to clarify the potential functional roles of different CLDN protein in CRC. Molecular docking analyses were used to probe potential interactions between CLDN4 and TGFβ1. Levels of CLDN4 and CLDN11 mRNA expression in clinical CRC patient samples and in the HT29 and HCT116 cell lines were assessed via qPCR. CLDN4 expression levels in these 2 cell lines were additionally assessed following TGFβ1 inhibitor treatment.

**Results:** These analyses revealed that COAD and READ tissues exhibited the upregulation of CLDN1, CLDN2, CLDN3, CLDN4, CLDN7, and CLDN12 as well as the downregulation of CLDN5 and CLDN11 relative to control tissues. Higher CLDN11 and CLDN14 expression as well as lower CLDN23 mRNA levels were associated with poorer overall survival (OS) outcomes. Moreover, CLDN2 and CLDN3 or CLDN11 mRNA levels were significantly associated with lymph node metastatic progression in COAD or READ lower in COAD and READ tissues. A positive correlation between the expression of CLDN11 and predicted macrophage, dendritic cell, and CD4^+^ T cell infiltration was identified in CRC, with CLDN12 expression further being positively correlated with CD4^+^ T cell infiltration whereas a negative correlation was observed between such infiltration and the expression of CLDN3 and CLDN15. A positive correlation between CLDN1, CLDN16, and neutrophil infiltration was additionally detected, whereas neutrophil levels were negatively correlated with the expression of CLDN3 and CLDN15. Molecular docking suggested that CLDN4 was able to directly bind via hydrogen bond with TGFβ1. Relative to paracancerous tissues, clinical CRC tumor tissue samples exhibited CLDN4 and CLDN11 upregulation and downregulation, respectively. LY364947 was able to suppress the expression of CLDN4 in both the HT29 and HCT116 cell lines.

**Conclusion:** Together, these results suggest that the expression of different CLDN family genes is closely associated with CRC tumor clinicopathological staging and immune cell infiltration. Moreover, CLDN4 expression is closely associated with TGFβ1 in CRC, suggesting that it and other CLDN family members may represent viable targets for antitumor therapeutic intervention.

## Introduction

Colorectal cancer (CRC) is the third most common and fourth deadliest cancer (Mármol et al., 2017). As CRC is often diagnosed at an early stage, it generally has a good 5-year overall survival (OS) rate, but patients diagnosed at a later stage with metastatic disease often fair poorly (Yamagishi et al., 2016). Diagnostic and prognostic biomarkers of CRC thus offer clear clinical value for identifying and monitoring affected patients.

Claudins (CLDNs) are members of a 27 + gene family of transmembrane proteins with four transmembrane helical domains, two extracellular loops, and short N- and C-terminal domains (Suzuki et al., 2014). These CLDNs play important roles in tumorigenesis and can influence aggressive growth and motility owing to their role as regulators of intercellular adhesion. Indeed, there is growing evidence that CLDN dysregulation is common across many cancer types such as gastric, lung, breast, ovarian, and colorectal cancer (Yu et al., 2009; Tabariès and Siegel, 2017).

Herein, we surveyed patterns of CLDN mRNA and protein expression in different CRC patient subsets in order to elucidate the links between these different genes and outcomes associated with different CRC stages. In addition, relationships between CLDN expression, mutational status, and immune cell infiltration in CRC tumors were assessed to better clarify the mechanistic role of these CLDNs and to guide the selection of future targets for therapeutic intervention when treating patients with this cancer type.

## Materials and Methods

### Clinical Samples

CRC patient tumor and paracancerous tissues were collected from the First Department of General Surgery, Shijiazhuang People’s Hospital (Hebei Province, China), and Hebei Medical University Fourth Hospital (China). Pathologists confirmed the diagnosis and staging of all patients, and the Ethical Committee of Shijiazhuang People’s Hospital approved this study. All patients provided written informed consent to participate.

### Cell Culture

Human HT29 and HCT116 cell lines were grown in McCoy’s 5A media (Gibco, CA, USA) containing 10% FBS and penicillin/streptomycin at 37°C in a 5% CO₂ incubator (Yang et al., 2021).

### Oncomine Database Analysis of CLDN mRNA Profiles

The Oncomine database (http://www.oncomine.com) (Rhodes et al., 2004) was utilized to evaluate the expression of 21 different CLDN family members in 20 cancer types, using the following criteria for differential expression: *p* = 0.01, Fold-change > 1.5, gene rank ≤10%.

### GEPIA Analyses

The GEPIA database (http://gepia.cancer-pku.cn/) (Tang et al., 2017) was used to analyze colon adenocarcinoma (COAD) and rectal adenocarcinoma (READ) tissue samples in order to explore the relationships between different CLDN family members and key clinical outcomes including staging, overall survival (OS), and disease-free survival (DFS). Default GEPIA parameters were utilized for these analyses.

### Human Protein Atlas Analyses

IHC staining data pertaining to the protein level expression of different CLDN family members were assessed with the Human Protein Atlas database (https://www.proteinatlas.org/) in CRC patient tumors and normal tissue samples (Pontén et al., 2008).

### Immune Cell Infiltration Analysis

The association between different CLDN family members and the infiltration of immune cells into CRC tumors was assessed with the TIMER database (https://cistrome.shinyapps.io/timer/), with scatter plots for different CLDN genes being generated to demonstrate purity-corrected partial Spearman’s r values and corresponding significance metrics (Li et al., 2021).

### UALCAN Database Analyses

Staging and nodal metastasis status for COAD (n = 324) and READ (n = 172) patient clinicopathologic parameters were evaluated using the UALCAN database (Lyu et al., 2020). According to multivariate Kaplan-Meier survival analysis, with *p*-values calculated using the log-rank test (log-rank test). The Ualcan database uses TCGA RNA-seq and clinical data for 31 cancer types.

### Analysis of CLDN Mutational Status in CRC

The mutational status of different CLDN family genes in CRC was assessed using the cBioPortal database (http://cbioportal.org) (Cerami et al., 2012). Assessed mutations included deep deletions, missense mutations, copy number amplifications, and mRNA upregulation.

### CLDN Matrix Interaction Analyses

An interaction matrix for CLDN family genes was generated using the “matrix” command in the GeneNetwork database (Mulligan et al., 2017), enabling a correlation analysis between different CLDN family members.

### Protein-Protein Interaction Network

The STRING database (http://string-db.org; version 11.0) was used to construct a CLDN family gene PPI network incorporating 24 co-expressed genes with a score of >0.4 (Franceschini et al., 2013). The network was visualized using Cytoscape (v 3.8.2).

### GO and KEGG Enrichment Analysis

GO analyses were used to assess the enrichment of particular genes in specific functional categories including biological processes (BPs), cellular components (CCs), and molecular functions (MFs), enabling efficient analyses of transcriptomic datasets (Thomas et al., 2019). The Kyoto Encyclopedia of Genes and Genomes (KEGG) database compiles functional data pertaining to a diverse array of regulatory pathways (Kanehisa et al., 2016). The online DAVID database (https://david.ncifcrf.gov/; version 6.8) was used for GO annotation and KEGG pathway analyses in this study (Huang et al., 2007). R was used to visualize the enrichment data.

### Molecular Docking Analysis

Full-length wild-type protein sequences were obtained from UniProt (https://www.uniprot.org/) (UniProt Consortium, 2021), with Uniprot ID. O14493 and Uniprot ID. P01137 being used for CLDN4 and TGFβ1, respectively. Three-dimensional structures for these proteins were obtained from the RCSB PDB database (https://www.rcsb.org/) (Berman et al., 2000), using PDB IDs of 7KP4 and 5VQP, respectively, for docking analyses. The CLDN4 and TGFβ1 proteins were docked with the Zdock server 3.0.2 (https://zdock.umassmed.edu/) (Pierce et al., 2014). Prior to docking, PyMOL was utilized to remove water molecules, heteroatoms, and repeated subunits, with the best docked complex being assessed for hydrogen bonding.

### qRT-PCR

The ISOGEN reagent (Nippon Gene Co. Ltd., Kokyo, Japan) was utilized to isolate RNA from tissue and cell line samples, after which qPCR was conducted by initially reverse transcribing 1 µg of total RNA per sample using a Revert Aid first strand cDNA synthesis kit (ThermoFisher Scientific). Primers used for qPCR are compiled in Supplementary Table S1. A Real-Time PCR system (BIOER Co. Ltd., Kokyo, Japan) was used for all qPCR analyses, and the relative expression of CLDN11, CLDN4, and TGFβ1 was assessed via the ΔCT method as in prior reports (Yang et al., 2021). For appropriate experiments, the HT29 and HCT116 cells were treated for 48 h with the TGFβ1 inhibitor LY364947 (5.00 or 10.0uM).

### Western Blot

HT29 and HCT116 cells were treated for 48 h with LY364947 at concentrations of 5, and 10 μM. After being washed three times with PBS, the cells were lysed for 30 min on ice before being centrifuged at 10000 g for 5 min at 4°C for 5 min. The protein concentrations in cell lysates were determined using an ND-1000 Spectrophotometer (National Instruments) (NanoDrop, ThermoFisher Scientific). SDS-PAGE was used to separate out equal amounts of total protein, which was then transferred to PVDF membranes (Millipore, Billerica, MA, USA). After blocking with 5% milk for 2 h, we did overnight immunoblotting with primary antibodies at 4°C. The primary antibodies were raised against TGFβ1 (1:1,000; Abcam, Cambridge, MA, USA), CLDN4 (1:500; abways technology) and β-actin (AC026, at 1 : 10000 dilution). A secondary antibody of goat anti-rabbit IG (KPL074-1,506, at 1: 5,000 dilution) was then used to incubate the membranes for 1 h at 37°C. Fusion FX5 Spectra was used to determine the intensity of protein bands (Fusion, France). The membranes were then incubated for 1 h at 37°C with secondary antibodies of goat anti-rabbit IG (KPL074-1,506, at 1:5,000 dilution). The intensity of protein bands was estimated via executing Fusion FX5 Spectra (Fusion, France).

## Results

### The Association Between CLDN Family Gene Expression and CRC Pathological Type

**FIGURE 1:**
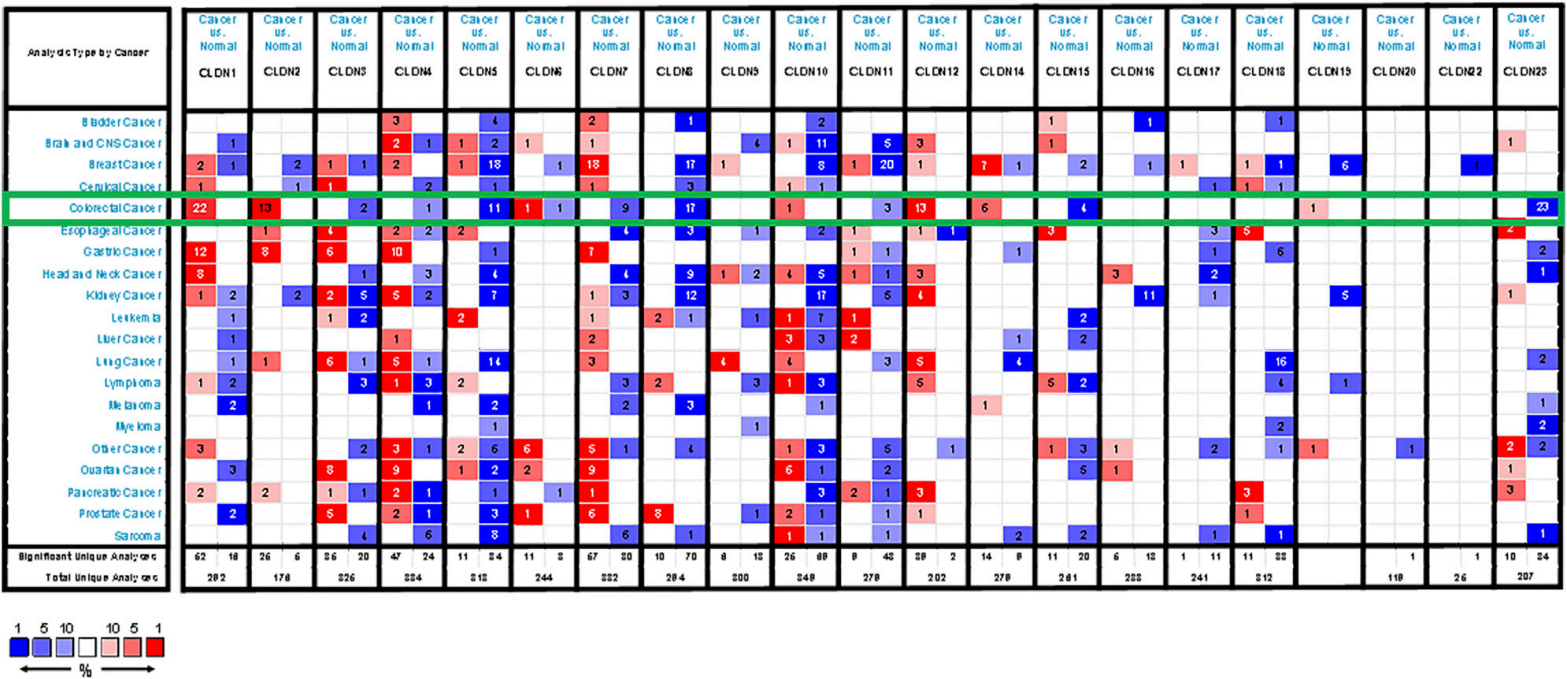
CLDN family member expression levels across human cancers. Cut-off values for these mRNA analyses were as follows: *p*-value = 0.01, Fold-change = 1.5, gene rank = 10%. Red and blue correspond to overexpression and underexpression, respectively, with the strength of the color be proportional to the expression level for that gene.

We began by using the Oncomine database to evaluate CLDN family member expression in CRC. In total, 19 CLDNs were found to be differentially expressed between normal tissues and COAD and READ patient samples. At the mRNA level, CLDN1, CLDN2, CLDN12, and CLDN14 were upregulated whereas CLDN3, CLDN5, CLDN7, CLDN8, CLDN11, CLDN15, and CLDN23 were downregulated (FC > 1.5) in patients with CRC (Figure 1). Specifically, fold-change values for CLDN1, CLDN2, and CLDN12 in COAD tissues were 6.755, 3.006, and 2.096, respectively (Supplementary Table S1), while in READ tissues these respective fold-change values were 4.301, 1.849, and 1.583 in the dataset analysis of Kaiser et al. (2007). CLDN19 expression was altered by 1.117 and 1.158-fold in COAD and READ tissues from this same dataset, while CLDN20 expression was changed by 1.075- and 1.091-fold, respectively. Gaedcke et al. (2010) found fold-change values for the expression of CLDN1, CLDN10, CLDN11, CLDN15, and CLDN22 of 19.563, 1.583, 1.718, 1.417, and 1.05, respectively, in READ tissues. Skrzypczak et al. (2010) further reported CLDN1 and CLDN2 expression levels in CRC tissues that were altered by 6.351- and 7.754-fold, respectively. In the TCGA dataset, CLDN14 expression in READ and COAD was altered by 4.876-fold and 4.368-fold, respectively, while it was altered by 3.471-fold in the dataset generated by Gaedke et al. CLDN18 expression was altered by 1.298-fold and 1.184-fold in COAD data from TCGA and Kaiser et al., and by 1.216-fold and 1.25-fold in READ samples from these two respective data sources.

**FIGURE 2:**
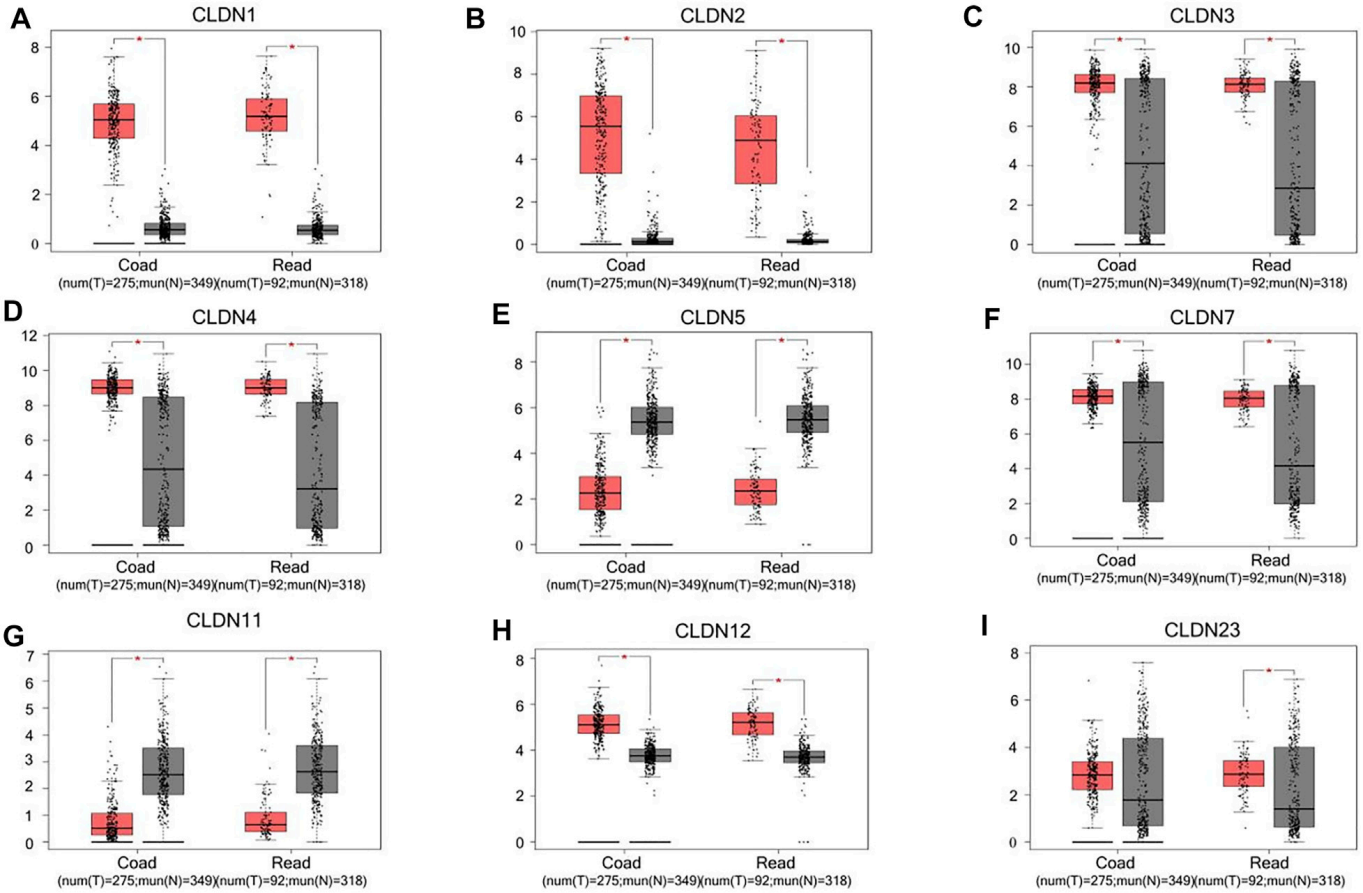
CLDN family gene expression in COAD and READ tissues. Tumors and normal tissue controls are respectively shown in red and grey **(A-I)**. *p < 0.05.

**FIGURE 3:**
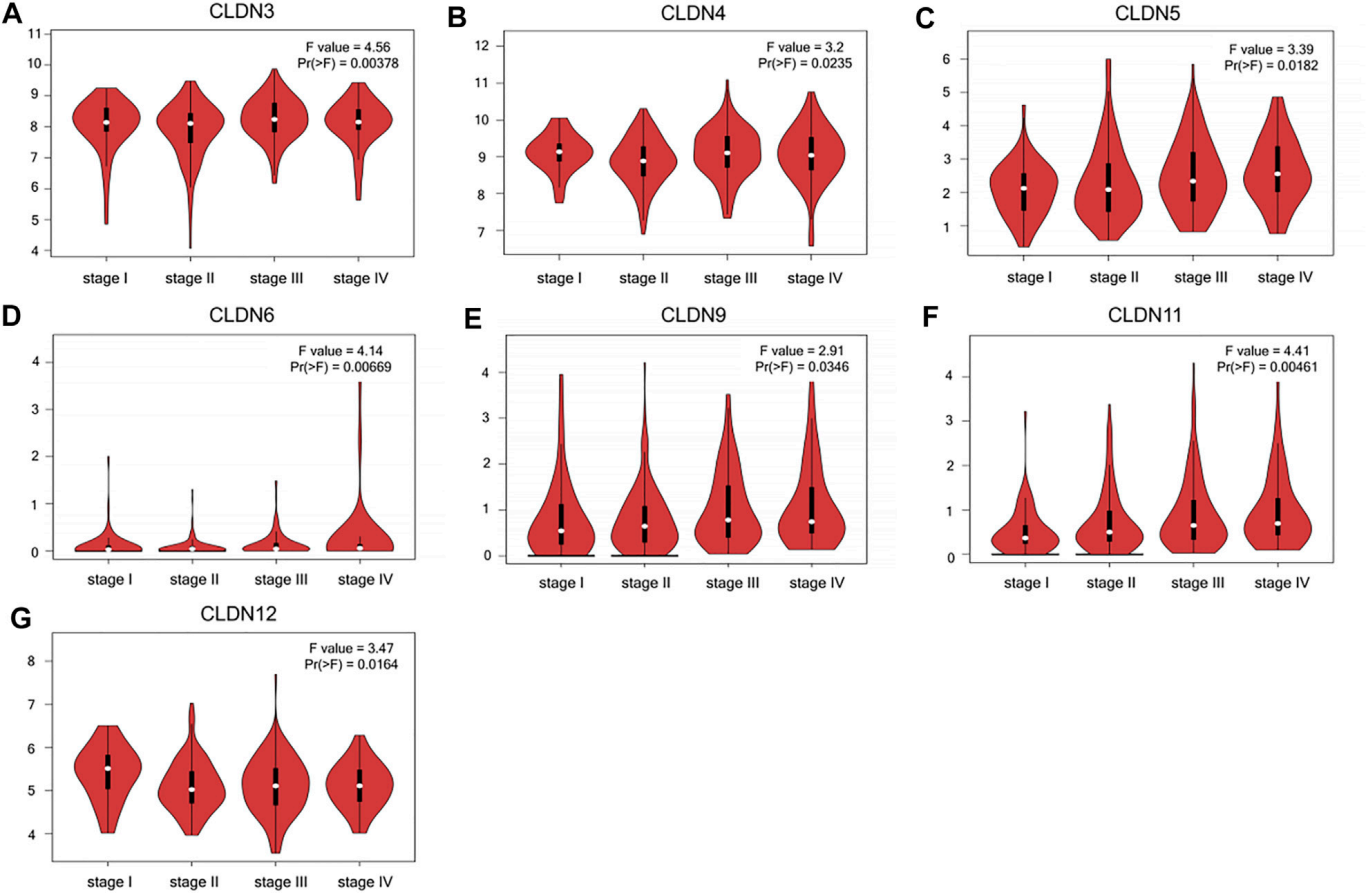
CLDN family gene expression as a function of CRC patient disease staging **(A-G)**.

**FIGURE 4:**
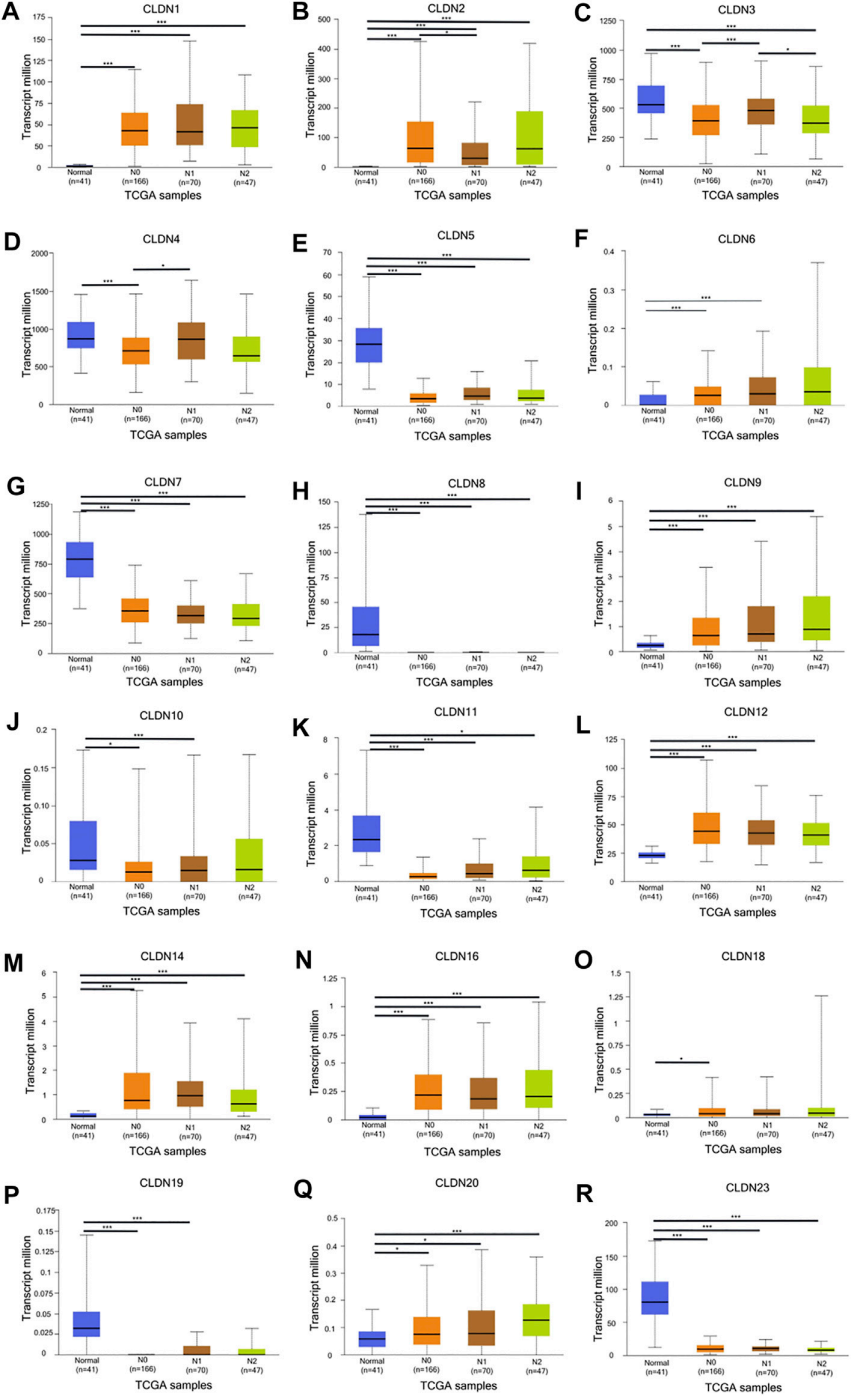
The UALCAN database shows a link between CLDN gene expression and nodal metastases in COAD patients. Box plots represent the CLDN mRNA expression levels in normal tissues or in COAD patients with N0–N2 disease **(A–R)**. *p < 0.05; **p < 0.01; ***p < 0.001.

**FIGURE 5:**
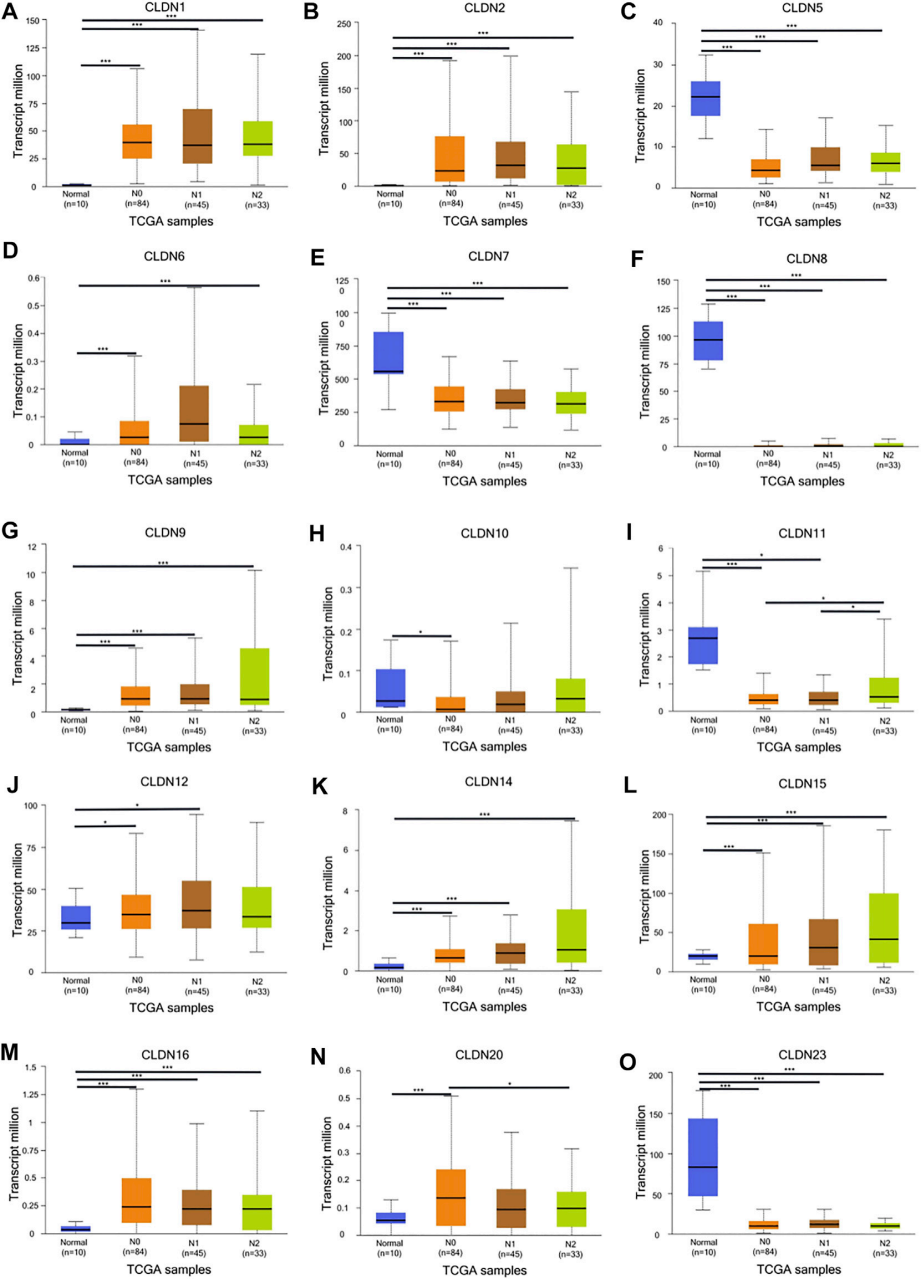
The UALCAN database shows a link between CLDN gene expression and nodal metastases in READ patients. Box plots represent the CLDN mRNA expression levels in normal tissues or in READ patients with N0–N2 disease **(A–O)**. *p < 0.05; **p < 0.01; ***p < 0.001.

The GEPIA database was next utilized to compare the expression of different CLDN family members in CRC and in normal colon tissue samples, revealing CLDN1, CLDN2, CLDN3, CLDN4, CLDN7, and CLDN12 to have been upregulated and CLDN5 and CLDN11 to have been downregulated in COAD and READ tissues relative to control samples (Figure 2). Significant differences in CLDN3, CLDN4, CLDN5, CLDN6, CLDN9, CLDN11, and CLDN12 expression were evident as a function of tumor stage (Figure 3). CLDN3, CLDN4 and CLDN6 were related with clinicopathological stage in CRC. The more CLDN5, CLDN9, and CLDN11 are expressed in CRC, the worse the stage. CLDN12 expression in CRC associated with pathological stage. We additionally examined the link between CLDN family gene mRNA levels and CRC patient lymph node metastasis, revealing a significant relationship between the expression of CLDN1, CLDN2, CLDN3, CLDN7, CLDN8, CLDN9, CLDN11, CLDN14, CLDN16 and CLDN23 nodal metastasis in COAD and READ (Figures 4A, B, C, G, H, I, K, M, N, R) (Figures 5A, B, C, E, F, G, I, K, M, O).

### The Association Between CLDN Expression and CRC Patient Survival Outcomes

**FIGURE 6:**
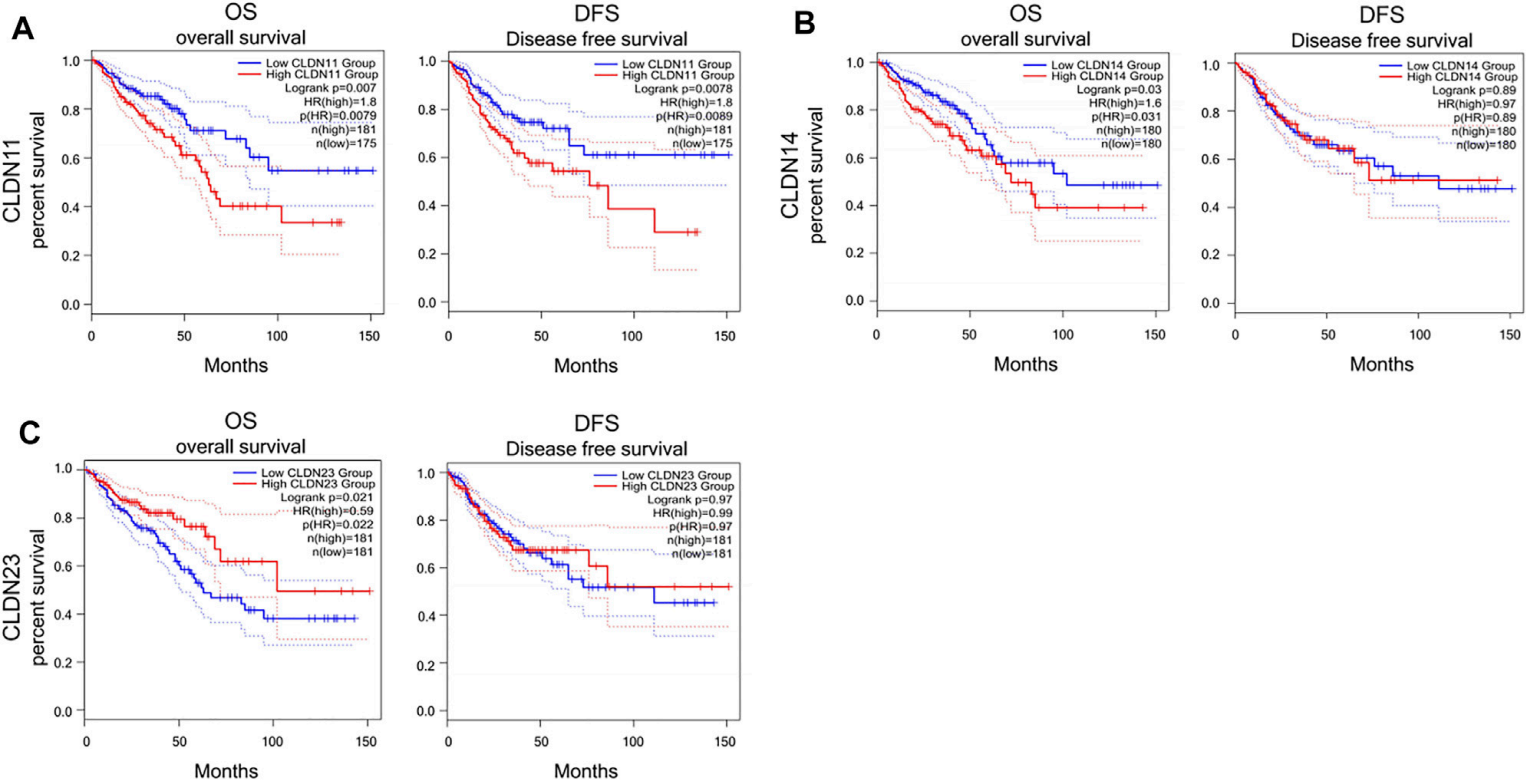
Kaplan–Meier survival analyses assing the link between CLDN expression and CRC patient survival in GEPIA database **(A-C)** (*p* < 0.05).

Kaplan-Meier curves and log-rank tests were next performed, revealing a relationship between the upregulation of CLDN11, CLDN14, and CLDN23 at the mRNA level and COAD and READ patient OS (Figure 6). Specifically, high levels of CLDN11 and CLDN14 expression were correlated with worse patient OS, whereas CLDN23 overexpression was linked to better OS outcomes.

### Analysis of CLDN Protein Levels in CRC

**FIGURE 7:**
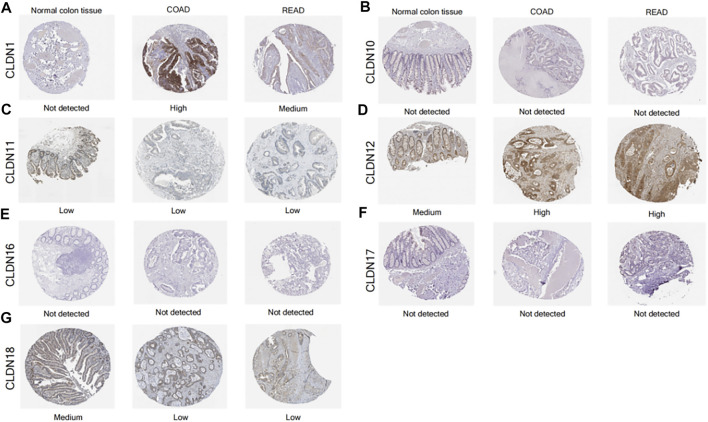
IHC staining for CLDN protein expression levels in tumor samples from CRC patients and normal colon tissue samples **(A-G)**.

The Human Protein Atlas yielded findings that were somewhat consistent with the mRNA level data above, with higher CLDN1 and CLDN12 protein levels and lower CLDN11 levels in CRC tumors relative to samples of normal colon tissue (Figure 7).

### The Relationship Between CLDN Expression and CRC Tumor Immune Cell Infiltration

**FIGURE 8:**
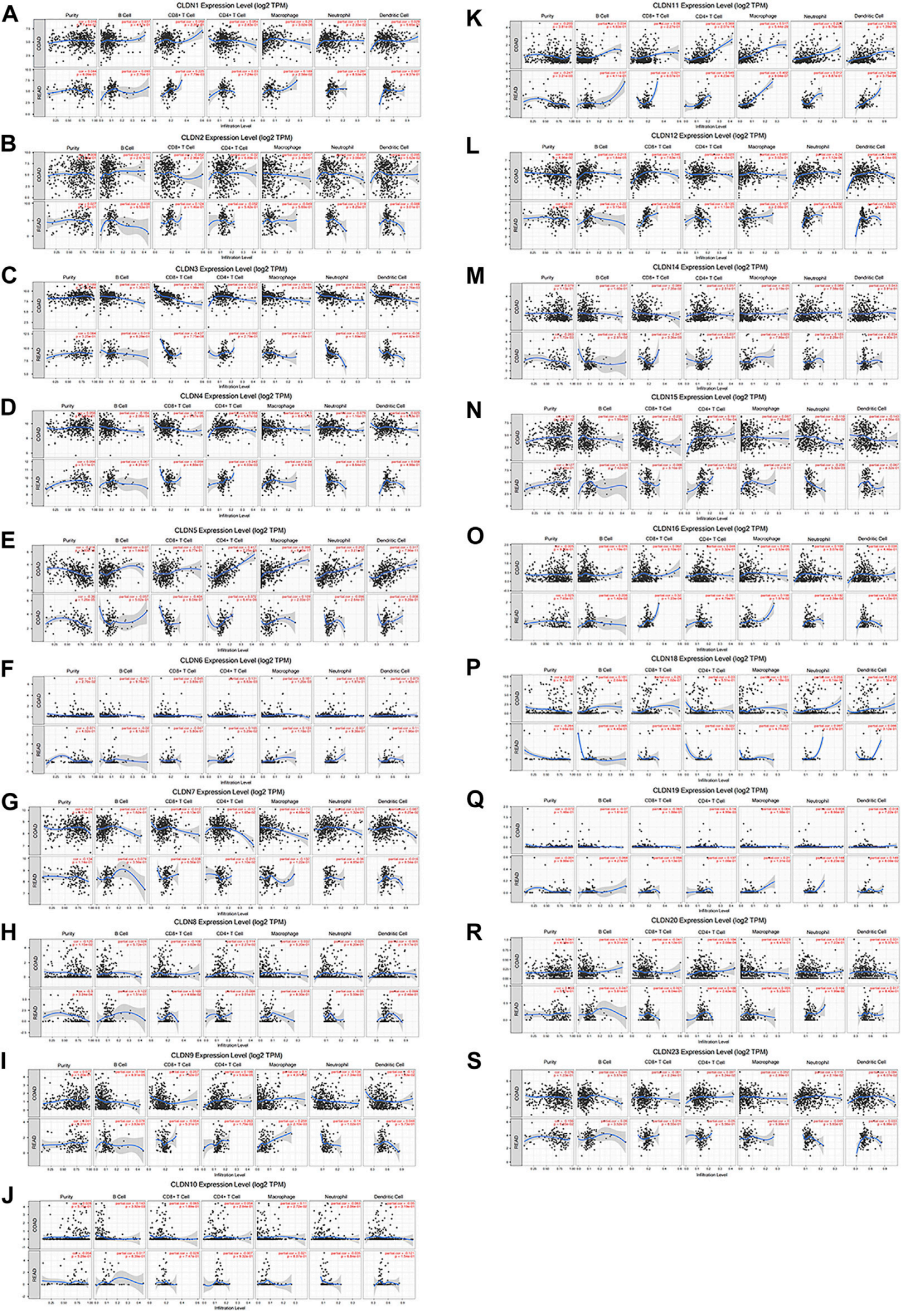
The relationship between CLDN mRNA level expression and CRC tumor immune cell infiltration **(A-S)**.

Using the TIMER database, we detected a negative correlation between the mRNA level expression of CLDN5, CLDN8, CLDN11, and CLDN18 and CRC tumor purity (Figures 8E, H, J, O). Moreover, CLDN12 expression was correlated with B cell infiltration and positively correlated with CD8^+^ T cells (Figures 8K). Additionally, CLDN5, CLDN9, CLDN11, CLDN15, and CLDN20 were significantly positively correlated with CD4^+^ T cell infiltration into CRC tumors, while CLDN7 expression was negatively correlated with such infiltration (Figures 8E, H, J, M, Q). Additionally, a clear positive correlation was observed between macrophage infiltration and the expression of CLDN1, CLDN9, CLDN11, and CLDN16 (Figures 8A,H,J,N). Neutrophil infiltration was negatively correlated with CLDN15 expression and positively correlated with CLDN1 and CLDN16 expression (Figures 8A, M, N), while CLDN11 expression was positively correlated with dendritic cell infiltration (Figures 8J).

### CLDN Mutation and the Relationships in CLDN Family in COAD Patients

**FIGURE 9:**
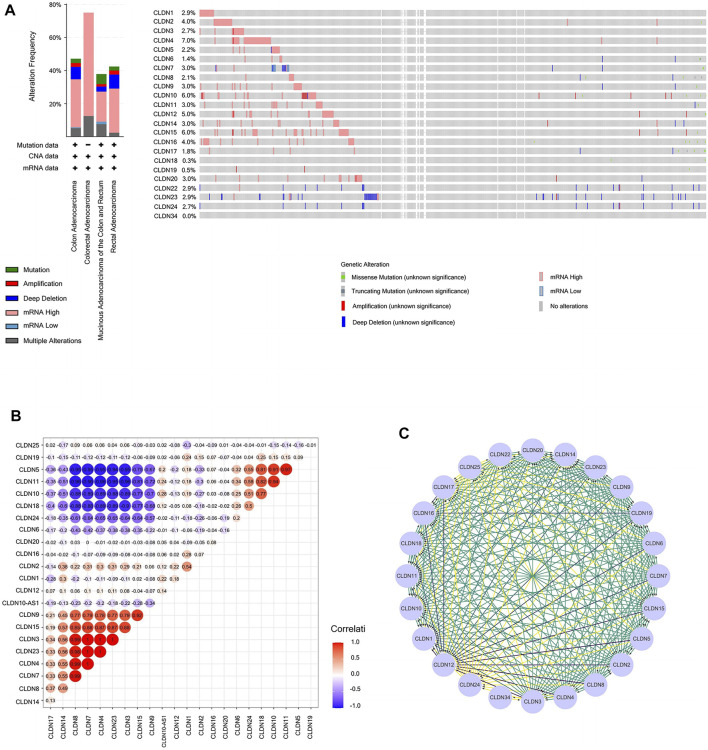
**(A)** Cancer type summary and Oncoprint for CLDN family genes in BioPortal. **(B)** Heatmap demonstrating correlations among CLDN family genes with respect to mRNA expression. Colors correspond to Pearson correlation coefficients, which span from blue to red (negative and positively correlated, respectively). **(C)** A PPI network of CLDNs was generated using the STRING database to clarify interactions among these proteins.

Next, we assessed CLDN family gene mutations in 640 COAD samples using the online cBioPortal tool, revealing mutations in 283 samples (44%) (Figures 9A). Mutations in CLDN10 (6%) were attributable to missense mutations and gene amplification.

The Genenetwork database was also used to explore relationships among CLDN gene expression levels in COAD samples, revealing the following correlative relationships: CLDN1 with CLDN2; CLDN3 with CLDN4, CLDN7, CLDN8, CLDN14, and CLDN23; CLDN4 with CLDN7, CLDN8, and CLDN14; CLDN5 with CLDN10, CLDN11, CLDN18, and CLDN24; CLDN7 with CLDN8 and CLDN14; CLDN8 with CLDN14; CLDN9 with CLDN3, CLDN4, CLDN7, CLDN8, CLDN14, CLDN15, and CLDN23; CLDN10 with CLDN18 and CLDN24; CLDN11 with CLDN10, CLDN18, and CLDN24; CLDN15 with CLDN3, CLDN4, CLDN7, CLDN8, CLDN14, and CLDN23; CLDN18 with CLDN24; and CLDN23 with CLDN4, CLDN7, CLDN8, CLDN14, and CLDN23. A co-expression network for these CLDNs is shown in Figures 9B.

A PPI network for these CLDN family genes was generated composed of 24 nodes and 254 edges (Figures 9C).

### GO Function and KEGG Pathway Enrichment Analyses of CLDN Genes in CRC

**FIGURE 10:**
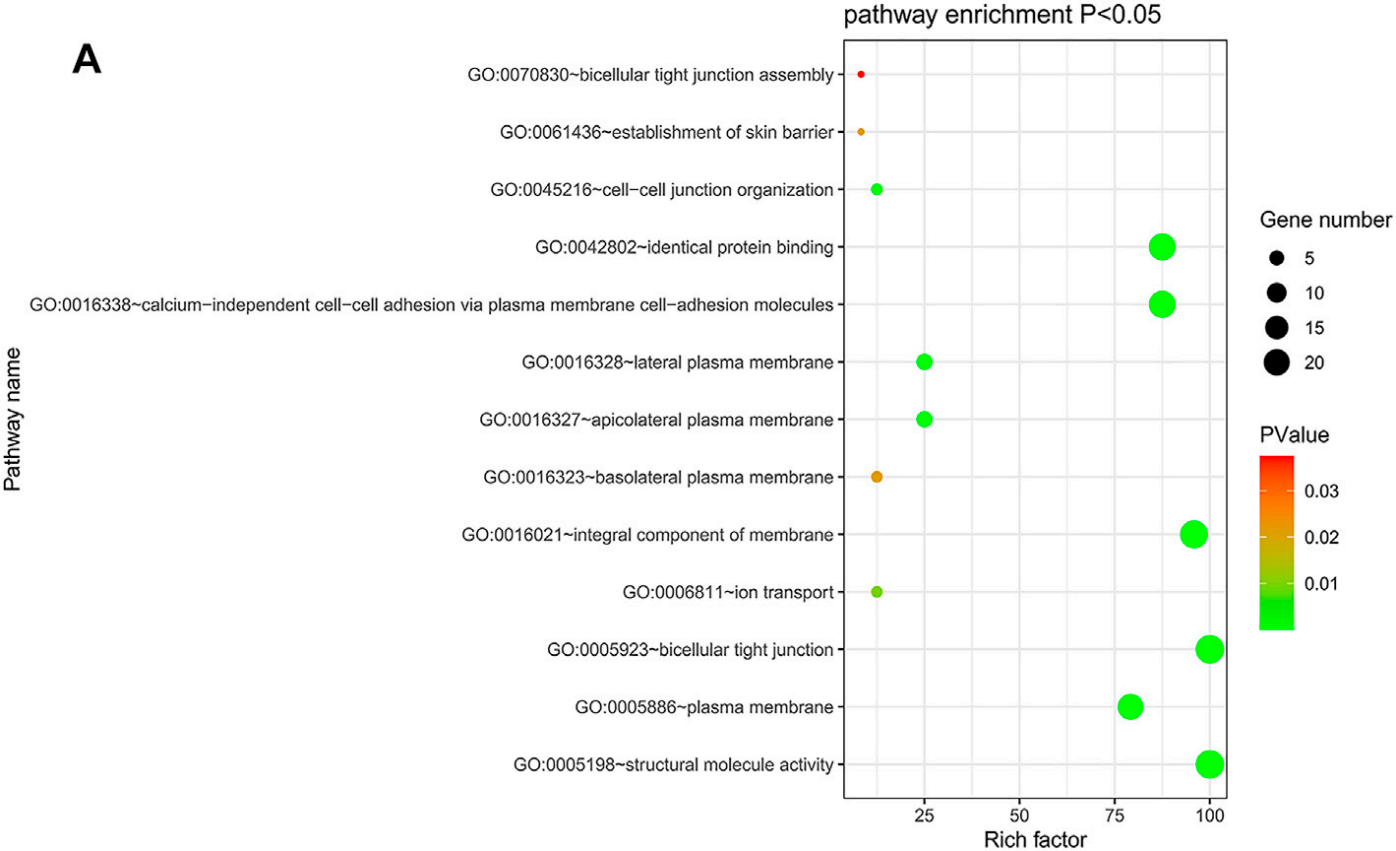
**(A)** DAVID was used for the functional enrichment analysis of CLDN family genes, with results shown using a bubble chart.

The DAVID database was utilized to predict the functional roles of different CLDNs in key CRC-related biological processes, molecular functions, and cellular components (Figures 10A). KEGG pathway analyses further revealed a close relationship between these CLDN family members and tight junctions, cell adhesion molecules, leukocyte transendothelial migration, and Hepatitis C (Supplementary Table S2).

### Molecular Docking Analyses

**FIGURE 11:**
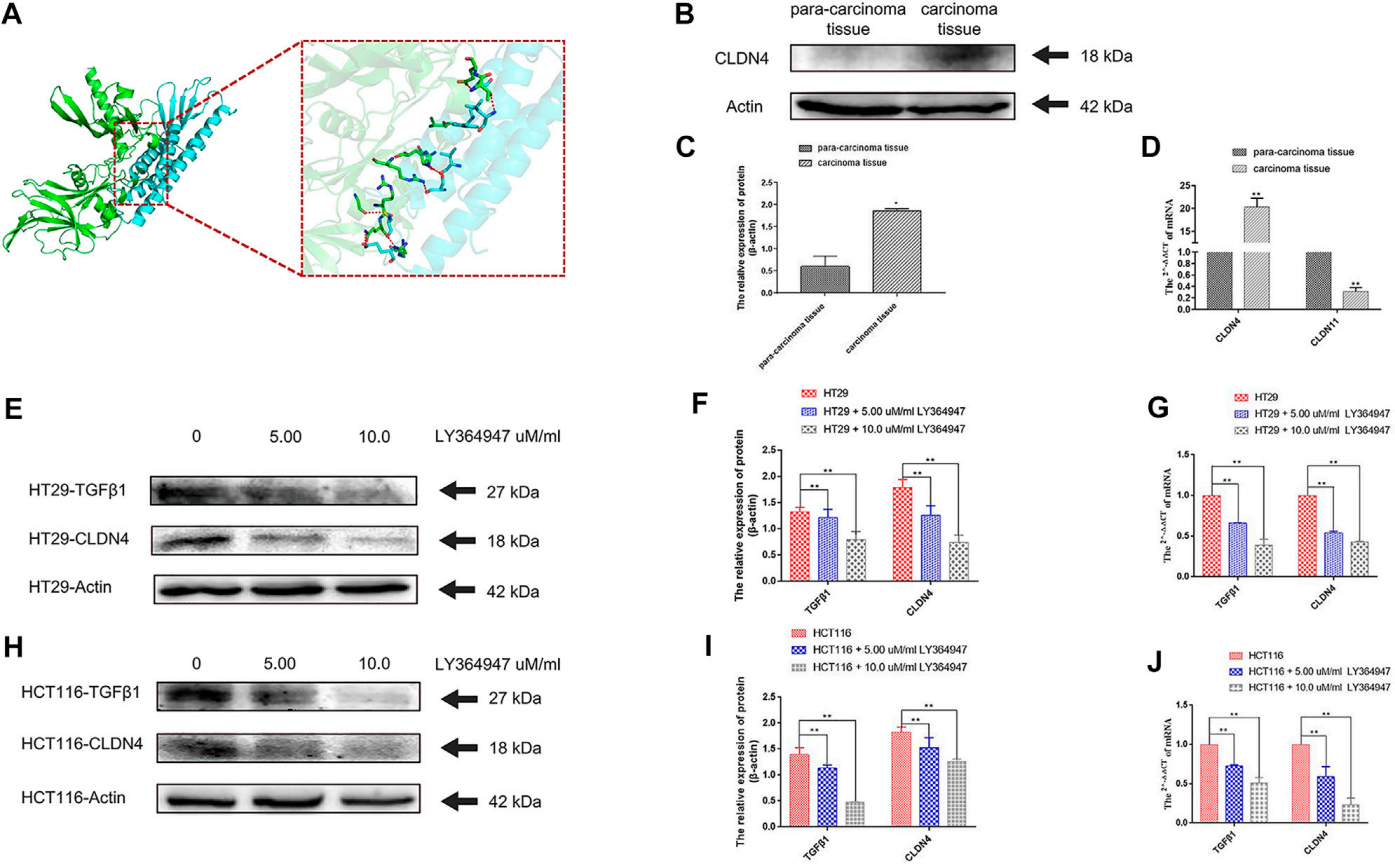
**(A)** A docking analysis of CLDN4 and TGFβ1. **(B) (C)** The protein of CLDN4 levels was increased in human CRC tissues (n = 5, *p < 0.05; **p < 0.01). **(D)** CLDN4 and CLDN11 mRNA levels were respectively increased and decreased in human CRC tissues (n = 10, *p < 0.05; **p < 0.01). **(E) (F) (G)** Western blot and qRT-PCR showed TGFβ1 and CLDN4 were remarkably downregulated at both protein and mRNA levels after treatment with LY364947 at 5.0 and 10.0 uM/ml, respectively, on HT29 cells. **(H) (I) (J)** Western blot and qRT-PCR showed TGFβ1 and CLDN4 were remarkably downregulated at both protein and mRNA levels after treatment with LY364947 at 5.0 and 10.0 uM/ml, respectively, on HCT116 cells. TGFβ1 reduced CLDN4 expression in colorectal cancer cell lines.

A prior study of inflammatory bowel disease demonstrated the ability of TGFβ1 signaling inhibition to suppress CLDN4 expression (Marincola Smith et al., 2021). A molecular docking analysis was therefore conducted to explore putative interactions between CLDN4 and TGFβ1. For this approach, ZDOCK scores were used to estimate protein-protein binding affinity, with higher scores being indicating of greater binding affinity (Pierce et al., 2011). This approach revealed that nine hydrogen bonds were predicted to form between CLDN4 and TGFβ1, with interactions between Glu109 of CLDN4 and Gln177 of TGFβ1, Cys107 of CLDN4 and Gly173 and Arg181 of TGFβ1, Val95 of CLDN4 rand Ser244 of TGFβ1, Ser98 of CLDN4 and His247 and Arg249 of TGFβ1, Trp18 of CLDN4 and Leu242 of TGFβ1, Ala20 of CLDN4 and Glu261 of TGFβ1, and Leu173 of CLDN4 and Thr260 of TGFβ1 (Figures 11A).

### Assessment of CLDN4 and CLDN11 Expression and the Responsiveness of CLDN4 to TGFβ1 Inhibitor Treatment

At the mRNA level, we found that CLDN11 and CLDN4 expression were respectively reduced and increased in CRC tumor samples relative to paracancerous controls as measured via qPCR (n = 10, *p* < 0.05). At the protein level, CLDN4 expression was increased by WB (n = 5, *p* < 0.05) (Figures 11B,C,D) (Supplementary Table S3). Treatment with LY364947 (TGFβ1 inhibitor) at a dose of five or 10 uM was sufficient to suppress CLDN4 mRNA and protein expression at 48 h post-treatment in both HT29 and HCT116 cells (Figure 11E, F, G, H, I, J).

## Discussion

Claudin (CLDN) genes encode a family of proteins that play a critical role in the generation and functioning of tight junctions between epithelial and endothelial cells (Hewitt et al., 2006). Many malignancies rely on this protein family for their development, progression, and metastasis. Although some studies have demonstrated the importance of claudins in tumorigenesis and the prognosis of human cancers (Arabzadeh et al., 2007; Morel et al., 2012), no extensive bioinformatic analyses of this gene family have been performed in colorectal cancer to date. In this study, each gene in the claudin family was evaluated to examine its expression and prognostic relevance in colorectal cancer using bioinformatics tools, offering an opportunity to better understand how claudin gene dysregulation affects colorectal cancer, as well as how treatment can be optimized to improve patient prognosis.

Our findings revealed that the mRNA expression levels of CLDN1, CLDN2, CLDN12, and CLDN14 were upregulated in colorectal cancer tissues relative to normal tissues in the Oncomine database, whereas CLDN3, CLDN5, CLDN7, CLDN8, CLDN11, CLDN15, and CLDN23 were downregulated, as previously reported. However, when comparing colorectal cancer tissues from the GEPIA database to normal colonic mucosa, the mRNA level expression of CLDN3, CLDN7, and CLDN23 was shown to be significantly higher in these tumor tissues. Given the inconsistencies in claudin gene expression in these two databases, we focused on those claudins exhibiting comparable gene expression patterns in both databases. In addition, three genes were shown to be related with advanced colorectal cancer: CLDN11 (*p* = 0.007), and CLDN14 (*p* = 0.03) and CLDN23 (*p* = 0.021). Moreover, we found that CLDN11 and CLDN18 protein levels were decreased in CRC tumors relative to normal tissues. At the same time, high levels of CLDN11 expression were associated with an increase in the proportion of CD4^+^ T cells, macrophages, neutrophils, and dendritic cells within tumors. We further determined that CLDN4 and TGFβ1 were capable of forming hydrogen bonds, and that CLDN4 was overexpressed in CRC cell lines and inhibited by TGFβ inhibitor treatment.

Claudin expression is related to tumor proliferation, migration, and invasion (Mori et al., 2011; Pope et al., 2014; Wang and Zöller, 2019; Li et al., 2020a). Many claudins are dysregulated in a range of cancers, as in the case of the CLDN1 and CLDN2 genes, which are overexpressed in colorectal cancer (Dhawan et al., 2011; Mori et al., 2011; Pope et al., 2014; Wang et al., 2019). CLDN3 is similarly overexpressed at the mRNA level in CRC tumors that belong to the consensus molecular subgroup (CMS)-CMS2 and CMS3, which correspond to a poor prognosis (Tang et al., 2011; Perez et al., 2020). Colorectal cancers have significant levels of CLDN4 expression (Georges et al., 2012). It has been shown that combination treatment with 5-fluorouracil (FU) and an anti-CLDN4 extracellular domain antibody (4D3) enhances antitumor efficacy against CRC (Fujiwara-Tani et al., 2018). CLDN5 expression has been shown to be reduced in squamous cell carcinomas of the lung (Akizuki et al., 2017), cervical cancer (Zhu et al., 2015), hepatocellular carcinoma (Sakaguchi et al., 2008), oral squamous cell carcinoma (Phattarataratip and Sappayatosok, 2016), breast cancer (Escudero-Esparza et al., 2012), and pancreatic cancer (Jakab et al., 2011). Similarly, compared to normal mucosal tissues, CLDN5 expression was downregulated in CRC tumors (Cherradi et al., 2019), although the mechanistic basis for this change remains poorly understood. CLDN6 is a cellular adhesion protein that is abundantly expressed in gastric tumors tissues and cell lines, and is associated with a poor prognosis. CLDN6 enhances gastric cancer cell proliferation and invasiveness via the YAP1 and YAP1-Snail1 axis (Li et al., 2020a). High expression of CLDN7 has been shown to accelerate pancreatic and colon cancer progression (Mu et al., 2019; Wang and Zöller, 2019). Cancer initiation cells (CICs) and nasopharyngeal carcinoma metastases in gastrointestinal tumors can be detected using CLDN7 as a marker protein (Liu et al., 2017; Kyuno et al., 2019). The MAPK/ERK signaling pathway is activated in colorectal cancers when CLDN8 is overexpressed (Cheng et al., 2019). In contrast, low levels of CLDN8 expression slow the progression of renal clear cell carcinoma via the EMT/AKT signaling pathway (Zhu et al., 2020). Colonic epithelial barrier function in mice is influenced by TGF family signaling, which regulates CLDN2, CLDN4, CLDN7, and CLDN8 in colonic epithelial cells. We additionally confirmed that TGFβ1 and CLDN4 engage in molecular structural interactions and that TGFβ1 inhibits CLDN4 transcription and protein expression in human colorectal cancer cell lines. Increased levels of TGFβ1 may affect the expression of CLDN4, potentially influencing CRC development. CLDN9 can be employed as a prognostic biomarker for gastric and esophageal cancer (Kang et al., 2020; Yu et al., 2020). CLDN10 overexpression promotes carcinogenesis in papillary thyroid cancer, which has a poor prognosis (Zhou et al., 2018). However, infiltration of B cells, CD8 + T cells, and macrophages as a consequence of increased CLDN10 expression improves the prognosis of patients with papillary thyroid carcinoma (Xiang et al., 2020). TGFβ or WNT/β-catenin-induced epithelial-mesenchymal transition (EMT) and tumor resistance may be linked to CLDN10, which offers promise as a prognostic biomarker in ovarian cancer (Gao et al., 2017; Li et al., 2020b). Hypermethylated CLDN11 can be utilized as a biomarker for the early detection of CRC melanoma, and gastric cancer (Agarwal et al., 2009; Karagiannis et al., 2014; Guo et al., 2019). While CLDN11 is perhaps the best-studied claudin in oncogenic contexts to date, it has yet to be identified as either a promoter or repressor of tumor growth (Bhat et al., 2020). CLDN12 is involved in the formation of Ca2+ channels in intestinal epithelial cells and is involved in Ca2+ uptake in intestinal epithelial cells (Fujita et al., 2008). According to prior studies, CLDN12 is the most uniformly expressed of these genes across murine organs (Hwang et al., 2014). By activating PI3K/AKT/mTOR, CLDN14 promotes CRC development (Qiao et al., 2021). CLDN15 has been shown to be a positive indicator associated with malignant pleural mesothelioma in clinical contexts (Watanabe et al., 2021), while CLDN16 is a predictor of oral squamous cell carcinoma, breast cancer, and thyroid cancer (Chen et al., 2010; Gomez-Rueda et al., 2016; Ribeiro et al., 2021). In gastric cancer, CLDN18 can be employed as a tumor marker (Matsusaka et al., 2016). Moreover, CRC patients with low levels of CLDN23 expression may benefit from its use as a prognostic, diagnostic, or therapeutic target (Oh et al., 2017; Liu et al., 2020).

Previous studies exploring the effects of CLDN1, CLDN2, CLDN4, CLDN11, CLDN12, CLDN14, and CLDN23 expression on CRC tumor growth have yielded findings consistent with our results in the present study. However, the influence of additional genes expressed in CRC on tumor growth remains unknown. In addition, as many of these studies are subject to various limitations, the same genes may not be consistently expressed in both databases analyzed in the present study, and the results may thus be skewed. Secondly, as we used an online database to analyze gene expression patterns, our findings remain to be verified in a large-scale colorectal cancer clinical study.

In conclusion, we herein found that many claudin family genes and proteins were dysregulated in CRC by querying multiple publicly available databases. Claudin family proteins have also been implicated in various phases of cancer development, lymph node metastasis, and immune cell infiltration. In addition, TGFβ1 was found to suppress the expression of CLDN4 in CRC cell lines. In general, claudin family genes contribute to the progression and prognosis of colorectal cancer, and they may thus be prospective targets for the development of novel pharmaceutical preparations capable of treating CRC.

## Data Availability

The datasets presented in this study can be found in online repositories. The names of the repository/repositories and accession number(s) can be found in the article/Supplementary Material.
