# Supplementary material for: Evaluation of the Prognostic Relevance of Differential Claudin Gene Expression Highlights Claudin-4 as Being Suppressed by TGFβ1 Inhibitor in Colorectal Cancer
Source: Front Genet. 2022 Feb 24;13:783016. doi: 10.3389/fgene.2022.783016 (PMC8907593; doi:10.3389/fgene.2022.783016)
Supplement: Supplementary file 3 [file Table1.DOCX]

# Table 1: Changes in Claudin family gene expression at the transcriptional level in different colorectal cancers and colon tissue samples from the Oncomine database.

| **Gene** | **Dataset** | **Normal (case)** | **Tumor (case)** | **Fold change** | **T-test** | **p-value** |
| --- | --- | --- | --- | --- | --- | --- |
| CLDN1 | Skrzypczak | Colon (24) | Colorectal carcinoma (36) | 16.039 | 21.29 | 1.66E-28 |
|  |  |  | Colorectal Adenocarcinoma (45) | 6.351 | 14.489 | 3.61E-21 |
|  | TCGA | Colon (19) Rectum (3) | Rectal Mucinous Adenocarcinoma (6) | 22.267 | 21.04 | 8.63E-16 |
|  |  |  | Rectal Adenocarcinoma (60) | 10.911 | 22.031 | 9.43E-35 |
|  |  |  | Colon Mucinous Adenocarcinoma (22) | 18.723 | 17.426 | 4.29E-21 |
|  |  |  | Cecum Adenocarcinoma (22) | 23.27 | 15.938 | 9.54E-19 |
|  |  |  | Colon Adenocarcinoma (101) | 10.897 | 26.032 | 3.17E-36 |
|  |  |  | Rectosigmoid Adenocarcinoma (3) | 22.417 | 12.581 | 3.67E-04 |
|  | Gaedcke | Rectum (65) | Rectal Adenocarcinoma (65) | 19.563 | 35.641 | 4.02E-59 |
|  | Skrzypczak 2 | Colon (10) | Colon Carcinoma Epithelia (5) | 22.75 | 47.898 | 1.11E-15 |
|  |  |  | Colon Adenoma (5) | 24.619 | 24.459 | 6.94E-11 |
|  |  |  | Colon Carcinoma (5) | 33.468 | 28.899 | 1.40E-12 |
|  |  |  | Colon Adenoma Epithelia (5) | 13.918 | 30.148 | 8.32E-10 |
|  | Kaiser | Colon (5) | Rectosigmoid Adenocarcinoma (10) | 9.956 | 13.357 | 2.95E-09 |
|  |  |  | Rectal Mucinous Adenocarcinoma (4) | 8.395 | 14.928 | 1.71E-06 |
|  |  |  | Cecum Adenocarcinoma (17) | 8.722 | 17.408 | 6.52E-10 |
|  |  |  | Colon Adenocarcinoma (41) | 6.755 | 14.623 | 1.62E-10 |
|  |  |  | Colon Mucinous Adenocarcinoma (13) | 4.807 | 7.208 | 1.22E-06 |
|  |  |  | Rectal Adenocarcinoma (8) | 4.301 | 4.688 | 7.17E-04 |
|  | Hong | Colon (12) | Colorectal Carcinoma (70) | 18.857 | 25.707 | 1.43E-19 |
| CLDN2 | Skrzypczak | Colorectal  Tissue (24) | Colorectal Adenocarcinoma (45) | 7.754 | 20.85 | 1.67E-29 |
|  |  |  | Colorectal Carcinoma (36) | 4.162 | 8.821 | 3.22E-11 |
|  | Skrzypczak 2 | Colon (10) | Colon Adenoma Epithelia (5) | 7.242 | 17.333 | 1.37E-07 |
|  |  |  | Colon Adenoma (5) | 12.685 | 17.698 | 3.57E-06 |
|  | TCGA | Colon (19) Rectum (3) | Cecum Adenocarcinoma (22) | 14.245 | 9.547 | 2.28E-12 |
|  |  |  | Rectal Adenocarcinoma (60) | 12.259 | 10.535 | 4.05E-14 |
|  |  |  | Colon Adenocarcinoma (101) | 11.221 | 10.805 | 1.56E-13 |
|  |  |  | Colon Mucinous Adenocarcinoma (22) | 5.685 | 6.447 | 1.69E-07 |
|  | Kaiser | Colon (5) | Colon Adenocarcinoma (41) | 3.006 | 6.705 | 1.74E-08 |
|  |  |  | Cecum Adenocarcinoma (17) | 5.3 | 6.208 | 4.12E-06 |
|  | Hong | Colon (12) | Colorectal Carcinoma (70) | 5.825 | 6.651 | 8.67E-08 |
|  | Gaedcke | Rectum (65) | Rectal Adenocarcinoma (65) | 2.072 | 8.596 | 1.11E-12 |
| CLDN3 | Kaiser | Colon (5) | Rectal Mucinous Adenocarcinoma (4) | 1.367 | 2.234 | 0.031 |
|  | Zou | Colon (8) | Colon Carcinoma (9) | 1.183 | 2.986 | 0.005 |
| CLDN4 | Ki | Colon (28)  Liver (13) | Colon Adenocarcinoma (50) | 1.861 | 3.676 | 2.63E-04 |
|  | TCGA | Colon (19) Rectum (3) | Rectosigmoid Adenocarcinoma (3) | 1.205 | 2.807 | 0.007 |
|  | Kaiser | Colon (5) | Rectal Mucinous Adenocarcinoma (4) | 1.969 | 3.247 | 0.009 |
|  | Skrzypczak 2 | Colon (10) | Colon Carcinoma (5) | 1.278 | 2.109 | 0.027 |
|  |  |  | Colon Carcinoma (5) | 1.135 | 2.076 | 0.03 |
| CLDN5 | TCGA | Colon (19) Rectum (3) | Colon Mucinous Adenocarcinoma (22) | 1.206 | 1.763 | 0.043 |
| CLDN6 | TCGA | Colon (19) Rectum (3) | Rectosigmoid Adenocarcinoma (3) | 3.526 | 15.174 | 9.07E-14 |
|  |  |  | Rectal Mucinous Adenocarcinoma (6) | 3.034 | 4.902 | 0.001 |
|  |  |  | Rectal Adenocarcinoma (60) | 1.879 | 5.89 | 1.16E-07 |
|  |  |  | Colon Adenocarcinoma (101) | 1.715 | 5.595 | 6.99E-07 |
|  |  |  | Cecum Adenocarcinoma (22) | 1.946 | 4.085 | 1.32E-04 |
|  |  |  | Colon Mucinous Adenocarcinoma (22) | 1.469 | 2.935 | 3.00E-03 |
|  | Gaedcke | Rectum (65) | Rectal Adenocarcinoma (65) | 1.073 | 2.818 | 0.003 |
|  | Skrzypczak | Colorectal  Tissue (24) | Colorectal Adenocarcinoma (45) | 1.061 | 1.751 | 0.043 |
| CLDN7 | Gaspar | Intestinal  Mucosa (22) | Colorectal Adenoma (56) | 1.145 | 2.31 | 0.013 |
|  | Ki | Colon (28)  Liver (13) | Colon Adenocarcinoma (50) | 1.788 | 3.27 | 9.03E-04 |
| CLDN8 | NA | NA | NA | NA | NA | NA |
| CLDN9 | Kaiser | Colon (5) | Rectal Adenocarcinoma (8) | 1.4 | 4.147 | 0.004 |
|  |  |  | Rectal Mucinous Adenocarcinoma (4) | 1.389 | 3.715 | 0.004 |
|  |  |  | Rectosigmoid Adenocarcinoma (10) | 1.359 | 3.517 | 0.005 |
|  |  |  | Colon Mucinous Adenocarcinoma (13) | 1.368 | 3.643 | 0.004 |
|  |  |  | Colon Adenocarcinoma (41) | 1.309 | 3.442 | 0.01 |
|  |  |  | Cecum Adenocarcinoma (17) | 1.187 | 2.096 | 0.042 |
|  | Gaedcke | Rectum (65) | Rectal Adenocarcinoma (65) | 1.204 | 5.959 | 1.30E-08 |
|  | Skrzypczak | Colorectal  Tissue (24) | Colorectal Adenocarcinoma (45) | 1.138 | 2.817 | 0.004 |
|  | TCGA | Colon (19) Rectum (3) | Rectal Adenocarcinoma (60) | 1.12 | 2.013 | 0.025 |
| CLDN10 | Graudens | Colon (12) | Colorectal Carcinoma (18) | 1.9 | 4.884 | 2.27E-05 |
|  |  | Colon (19) Rectum (3) | Rectal Mucinous Adenocarcinoma (6) | 2.145 | 4.126 | 3.00E-03 |
|  |  |  | Colon Adenocarcinoma (101) | 1.539 | 5.011 | 2.22E-06 |
|  |  |  | Rectosigmoid Adenocarcinoma (3) | 5.916 | 3.674 | 0.028 |
|  |  |  | Rectal Adenocarcinoma (60) | 1.583 | 4.19 | 3.63E-05 |
|  |  |  | Cecum Adenocarcinoma (22) | 1.405 | 3.13 | 0.002 |
|  |  |  | Colon Mucinous Adenocarcinoma (22) | 1.225 | 2.055 | 0.023 |
|  | Skrzypczak 2 | Colon (10) | Colon Carcinoma (5) | 3.644 | 10.577 | 5.96E-05 |
|  |  |  | Colon Carcinoma Epithelia (5) | 2.125 | 5.148 | 0.002 |
|  | Skrzypczak | Colorectal  Tissue (24) | Colorectal Adenocarcinoma (45) | 1.268 | 3.048 | 0.002 |
|  |  |  | Colorectal Carcinoma (36) | 1.236 | 2.239 | 0.015 |
|  | Gaedcke | Rectum (65) | Rectal Adenocarcinoma (65) | 1.419 | 2.841 | 0.003 |
| CLDN11 | Gaspar | Intestinal  Mucosa (22) | Colorectal Adenoma (56) | 1.388 | 3.781 | 1.68E-04 |
|  | Gaedcke | Rectum (65) | Rectal Adenocarcinoma (65) | 1.718 | 7.151 | 4.54E-11 |
|  | Skrzypczak 2 | Colon (10) | Colon Carcinoma (5) | 1.303 | 4.694 | 6.87E-04 |
|  |  |  | Colon Carcinoma (5) | 2.016 | 3.868 | 0.005 |
|  | TCGA | Colon (19) Rectum (3) | Colon Mucinous Adenocarcinoma (22) | 1.209 | 1.802 | 0.04 |
| CLDN12 | Kaiser | Colon (5) | Cecum Adenocarcinoma (17) | 2.443 | 9.946 | 1.95E-09 |
|  |  |  | Colon Mucinous Adenocarcinoma (13) | 1.892 | 8.612 | 1.07E-07 |
|  |  |  | Colon Adenocarcinoma (41) | 2.096 | 13.812 | 3.00E-11 |
|  |  |  | Rectosigmoid Adenocarcinoma (10) | 1.904 | 7.573 | 3.27E-06 |
|  |  |  | Rectal Adenocarcinoma (8) | 1.849 | 6.098 | 9.76E-05 |
|  |  |  | Rectal Mucinous Adenocarcinoma (4) | 3.201 | 10.981 | 2.69E-04 |
|  | TCGA | Colon (19) Rectum (3) | Colon Mucinous Adenocarcinoma (22) | 2.217 | 10.308 | 2.05E-12 |
|  |  |  | Colon Adenocarcinoma (101) | 1.617 | 9.545 | 5.70E-13 |
|  | Skrzypczak | Colorectal  Tissue (24) | Colorectal Carcinoma (36) | 1.726 | 6.943 | 1.89E-09 |
|  |  |  | Colorectal Adenocarcinoma (45) | 1.636 | 6.746 | 3.47E-09 |
|  | Hong | Colon (12) | Colorectal Carcinoma (70) | 1.736 | 8.486 | 1.65E-09 |
|  | Skrzypczak 2 | Colon (10) | Colon Carcinoma (5) | 1.538 | 6.487 | 1.10E-05 |
| CLDN14 | TCGA | Colon (19) Rectum (3) | Colon Mucinous Adenocarcinoma (22) | 4.86 | 7.9 | 7.48E-10 |
|  |  |  | Cecum Adenocarcinoma (22) | 5.602 | 8.031 | 7.57E-10 |
|  |  |  | Colon Adenocarcinoma (101) | 4.876 | 9.655 | 1.13E-14 |
|  |  |  | Rectal Adenocarcinoma (60) | 4.368 | 7.846 | 1.26E-11 |
|  | Gaedcke | Rectum (65) | Rectal Adenocarcinoma (65) | 3.471 | 12.413 | 6.97E-21 |
|  | Kaiser | Colon (5) | Rectal Adenocarcinoma (8) | 1.583 | 4.94 | 2.84E-04 |
|  | Skrzypczak | Colorectal  Tissue (24) | Colorectal Carcinoma (36) | 1.192 | 3.937 | 1.20E-04 |
|  |  |  | Colorectal Adenocarcinoma (45) | 1.124 | 3.461 | 4.82E-04 |
|  | Skrzypczak 2 | Colon (10) | Colon Adenoma (5) | 1.147 | 3.658 | 0.002 |
|  | Hong | Colon (12) | Colorectal Carcinoma (70) | 1.642 | 2.007 | 0.032 |
| CLDN15 | Skrzypczak | Colon (10) | Colon Carcinoma (5) | 2.336 | 9.018 | 6.24E-06 |
|  |  |  | Colon Carcinoma Epithelia (5) | 1.24 | 2.42 | 1.80E-02 |
|  | Gaedcke | Rectum (65) | Rectal Adenocarcinoma (65) | 1.417 | 3.848 | 1.22E-04 |
| CLDN16 | Skrzypczak | Colorectal  Tissue (24) | Colorectal Carcinoma (36) | 1.241 | 4.27 | 4.13E-05 |
|  |  |  | Colorectal Adenocarcinoma (45) | 1.128 | 2.778 | 0.004 |
|  | Hong | Colon (12) | Colorectal Carcinoma (70) | 2.089 | 5.026 | 1.02E-05 |
|  | Gaedcke | Rectum (65) | Rectal Adenocarcinoma (65) | 1.064 | 4.431 | 1.14E-05 |
|  | TCGA | Colon (19) Rectum (3) | Cecum Adenocarcinoma (22) | 1.283 | 1.986 | 0.028 |
|  |  |  | Colon Adenocarcinoma (101) | 1.331 | 2.41 | 0.012 |
|  |  |  | Rectal Adenocarcinoma (60) | 1.333 | 2.244 | 0.016 |
| CLDN17 | TCGA | Colon (19) Rectum (3) | Rectal Mucinous Adenocarcinoma (6) | 1.402 | 4.629 | 8.25E-05 |
|  |  |  | Rectal Adenocarcinoma (60) | 1.281 | 3.526 | 4.80E-04 |
|  |  |  | Colon Adenocarcinoma (101) | 1.243 | 3.489 | 7.16E-04 |
|  |  |  | Colon Mucinous Adenocarcinoma (22) | 1.295 | 2.555 | 0.007 |
|  | Skrzypczak | Colorectal  Tissue (24) | Colorectal Adenocarcinoma (45) | 1.137 | 3.02 | 0.002 |
| CLDN18 | TCGA | Colon (19) Rectum (3) | Rectosigmoid Adenocarcinoma (3) | 1.163 | 2.478 | 0.011 |
|  |  |  | Colon Mucinous Adenocarcinoma (22) | 2.893 | 3.782 | 4.60E-04 |
|  |  |  | Rectal Mucinous Adenocarcinoma (6) | 1.203 | 2.162 | 2.00E-02 |
|  |  |  | Cecum Adenocarcinoma (22) | 1.488 | 2.568 | 0.008 |
|  |  |  | Colon Adenocarcinoma (101) | 1.298 | 2.896 | 0.003 |
|  |  |  | Rectal Adenocarcinoma (60) | 1.216 | 2.02 | 0.024 |
|  | Skrzypczak | Colon (10) | Colon Adenoma (5) | 1.228 | 3.557 | 0.002 |
|  | Kaiser | Colon (5) | Rectal Adenocarcinoma (8) | 1.205 | 3.035 | 0.007 |
|  |  |  | Colon Mucinous Adenocarcinoma (13) | 1.85 | 2.917 | 0.006 |
|  |  |  | Colon Adenocarcinoma (41) | 1.184 | 2.406 | 0.014 |
|  |  |  | Rectal Mucinous Adenocarcinoma (4) | 1.136 | 1.997 | 0.043 |
|  | Skrzypczak | Colorectal  Tissue (24) | Colorectal Adenocarcinoma (45) | 1.159 | 3.047 | 0.002 |
|  |  |  | Colorectal Carcinoma (36) | 1.299 | 2.152 | 0.019 |
|  | Hong | Colon (12) | Colorectal Carcinoma (70) | 1.745 | 2.61 | 0.008 |
| CLDN19 | Hong | Colon (12) | Colorectal Carcinoma (70) | 2.406 | 6.544 | 1.10E-07 |
|  | Kaiser | Colon (5) | Colon Adenocarcinoma (41) | 1.117 | 4.948 | 4.50E-05 |
|  |  |  | Rectal Adenocarcinoma (8) | 1.158 | 2.985 | 0.008 |
|  |  |  | Cecum Adenocarcinoma (17) | 1.126 | 3.196 | 0.002 |
|  |  |  | Colon Mucinous Adenocarcinoma (13) | 1.103 | 3.281 | 2.00E-03 |
|  | TCGA | Colon (19)  Rectum (3) | Rectosigmoid Adenocarcinoma (3) | 1.979 | 4.737 | 0.007 |
|  | Skrzypczak 2 | Colon (10) | Colon Adenoma (5) | 1.222 | 3.038 | 0.006 |
|  | Skrzypczak 2 | Colorectal  Tissue (24) | Colorectal Adenocarcinoma (45) | 1.103 | 3.281 | 0.001 |
|  |  |  | Colorectal Carcinoma (36) | 1.083 | 2.31 | 0.012 |
| CLDN20 | Kaiser | Colon (5) | Cecum Adenocarcinoma (17) | 1.132 | 3.206 | 0.006 |
|  |  |  | Rectal Mucinous Adenocarcinoma (4) | 1.117 | 2.826 | 0.013 |
|  |  |  | Rectal Adenocarcinoma (8) | 1.091 | 2.04 | 0.035 |
|  |  |  | Colon Mucinous Adenocarcinoma (13) | 1.084 | 2.05 | 0.036 |
|  |  |  | Colon Adenocarcinoma (41) | 1.075 | 1.98 | 0.045 |
|  | Skrzypczak 2 | Colon (10) | Colon Adenoma (5) | 1.171 | 2.092 | 0.043 |
| CLDN21 | NA | NA | NA | NA | NA | NA |
| CLDN22 | Gaedcke | Rectum (65) | Rectal Adenocarcinoma (65) | 1.05 | 5.625 | 6.36E-08 |
| CLDN23 | NA | NA | NA | NA | NA | NA |
| CLDN24 | NA | NA | NA | NA | NA | NA |
| CLDN25 | NA | NA | NA | NA | NA | NA |

# Table 2: KEGG pathway analysis of the CLDN family genes and similar co-expressed genes in CRC.

| Category | Term | Count | % | PValue | FDR |
| --- | --- | --- | --- | --- | --- |
| KEGG_PATHWAY | hsa04530:Tight junction | 22 | 91.666667 | 1.03E-41 | 2.06E-41 |
| KEGG_PATHWAY | hsa04670:Leukocyte transendothelial migration | 22 | 91.666667 | 7.16E-39 | 7.16E-39 |
| KEGG_PATHWAY | hsa05160:Hepatitis C | 22 | 91.666667 | 2.01E-37 | 2.01E-37 |
| KEGG_PATHWAY | hsa04514:Cell adhesion molecules (CAMs) | 22 | 91.666667 | 8.86E-37 | 8.86E-37 |

# Table 3. Primers used for this analysis

| Gene | Forward (5’-3’) | Reverse (5’-3’) |
| --- | --- | --- |
| β-actin | TGACGTGGACATCCGCAAAG | CTGGAAGGTGGACAGCGAGG |
| CLDN4 | TGGGGCTACAGGTAATGGG | GGTCTGCGAGGTGACAATGTT |
| CLDN11 | CGGTGTGGCTAAGTACAGGC | CGCAGTGTAGTAGAAACGGTTTT |
| TGFβ1 | CTAATGGTGGAAACCCACAACG | TATCGCCAGGAATTGTTGCTG |
